# Genetic and environmental factors regulate the type 1 diabetes gene *CTSH via* differential DNA methylation

**DOI:** 10.1016/j.jbc.2021.100774

**Published:** 2021-05-14

**Authors:** Jody Ye, Mihaela Stefan-Lifshitz, Yaron Tomer

**Affiliations:** Division of Endocrinology, Department of Medicine, Albert Einstein College of Medicine, Bronx, New York, USA

**Keywords:** DNA methylation, beta cell, genetic polymorphism, type 1 diabetes, epigenetics, ACTB, beta-actin, CpG, cytosine–phosphate–guanine dinucleotide, *CTSH*, cathepsin H, DMOG, dimethyloxalylglycine, DNMT, DNA methyltransferase, EF-1α, elongation factor 1α, GADD45α, growth arrest and DNA damage–45 alpha, GTEx, Genotype-Tissue Expression, GWAS, Genome-Wide Association Studies, HEK293, human embryonic kidney 293 cells, IFN-γ, interferon γ, IL-1β, interleukin 1β, LD, linkage disequilibrium, MS-qPCR, methylation-specific quantitative PCR, P/S, penicillin–streptomycin, T1D, type 1 diabetes, TDG, thymine DNA glycosylase, Tet, 10–11 translocation, TNF-α, tumor necrosis factor α, TSS, transcription start site

## Abstract

Cathepsin H (*CTSH*) is a type 1 diabetes (T1D) risk gene; large-scale genetic and epidemiological studies found that T1D genetic risk correlates with high *CTSH* expression, rapid decline of beta-cell function, and early onset T1D. Counterintuitively, transcriptional downregulation of *CTSH* by proinflammatory cytokines has been shown to promote beta-cell apoptosis. Here, we potentially explain these observed contrasting effects, describing a new mechanism where proinflammatory cytokines and T1D genetic risk variants regulate *CTSH* transcription *via* differential DNA methylation. We show that, in human islets, *CTSH* downregulation by the proinflammatory cytokine cocktail interleukin 1β + tumor necrosis factor α + interferon γ was coupled with DNA hypermethylation in an open chromatin region in *CTSH* intron 1. A luciferase assay in human embryonic kidney 293 cells revealed that methylation of three key cytosine–phosphate–guanine dinucleotide (CpG) residues in intron 1 was responsible for the reduction of promoter activity. We further found that cytokine-induced intron 1 hypermethylation is caused by lowered Tet1/3 activities, suggesting that attenuated active demethylation lowered *CTSH* transcription. Importantly, individuals who carry the T1D risk variant showed lower methylation variability at the intron 1 CpG residues, presumably making them less sensitive to cytokines, whereas individuals who carry the protective variant showed higher methylation variability, presumably making them more sensitive to cytokines and implying differential responses to environment between the two patient populations. These findings suggest that genetic and environmental influences on a T1D locus are mediated by differential variability and mean of DNA methylation.

Type 1 diabetes (T1D) is caused by loss of immune tolerance to insulin-producing beta cells in the pancreas. Beta-cell autoimmune destruction is believed to be triggered by certain environmental influences, such as microbial infections or dietary components (1). Subsequently, immune cells infiltrate the islets, causing beta-cell destruction, either directly or through secretion of proinflammatory cytokines, such as interleukin 1β (IL-1β), tumor necrosis factor α (TNF-α), and interferon γ (IFN-γ). The extent and rate of progression of beta-cell destruction are modulated by the individual's genetic background. It was shown that genes that frequently interact with environmental factors were enriched in disease risk loci (2, 3). In addition, these interactions can be mediated by epigenetic mechanisms, such as histone remodeling and cytosine–phosphate–guanine dinucleotide (CpG) methylation (4, 5). Epigenetically regulated loci thus can serve as environmental sensors that may help delineate mechanisms underlying the complex etiology of T1D. However, the mechanisms by which genes interact with environmental factors to trigger beta-cell autoimmunity are still unknown.

We have previously explored the potential epigenetic involvement in mediating T1D risk using causal-inference analyses (6, 7). We identified several susceptibility loci where DNA methylation likely mediates the risk of T1D; one of these susceptibility loci is the cathepsin H (*CTSH*) locus (8).

*CTSH* has been associated with T1D by Genome-Wide Association Studies (GWAS) (9, 10, 11, 12). Patients with the T1D risk variant correlated with increased *CTSH* transcription, early onset T1D (younger than 7 years), and rapid decline of beta-cell function (12, 13). Counterintuitively, functional studies demonstrated that the transcriptional downregulation of *CTSH* promoted beta-cell apoptosis (14). *CTSH* is silenced by proinflammatory cytokines, such as IL-1β, TNF-α, and IFN-γ (14). In addition, patients who carry the protectively variant exhibited lower *CTSH* expression, higher HbA1c, and less diabetes remission (14). We hypothesized that DNA methylation is involved in regulating the genetic and environmental influences of *CTSH* expression and that understanding its molecular mechanism will potentially unravel the observed paradox in T1D.

## Results

### Downregulation of *CTSH* expression by proinflammatory cytokines is accompanied by hypermethylation of a region in intron 1

**Figure 1:**
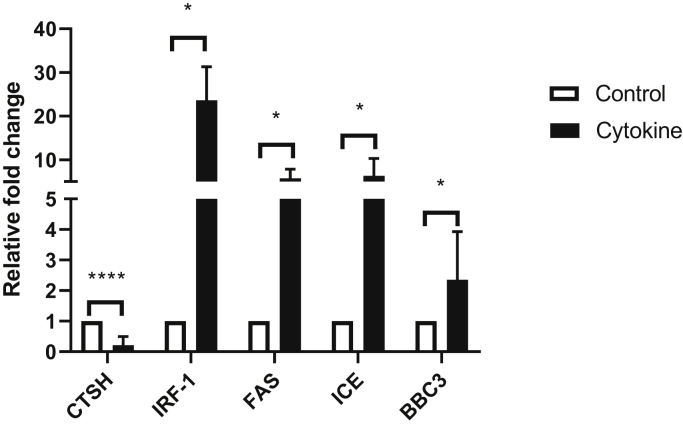
**Proinflammatory cytokines IL-1β + TNF-α + IFN-γ induces *CTSH* transcriptional downregulation and elevation of cytokine responsive genes.** Quantitative PCR analysis of transcription of *CTSH* (n = 10), *IRF-1* (n = 4), *FAS* (n = 4), *ICE* (n = 4), and *BBC3* (n = 4) in control and cytokine-treated human islets. Gene expression was normalized against beta-actin, and levels in control islets were arbitrarily set to one. *CTSH*, cathepsin H; IFN-γ, interferon γ; IL-1β, interleukin 1β; TNF-α, tumor necrosis factor α.

**Figure 2:**
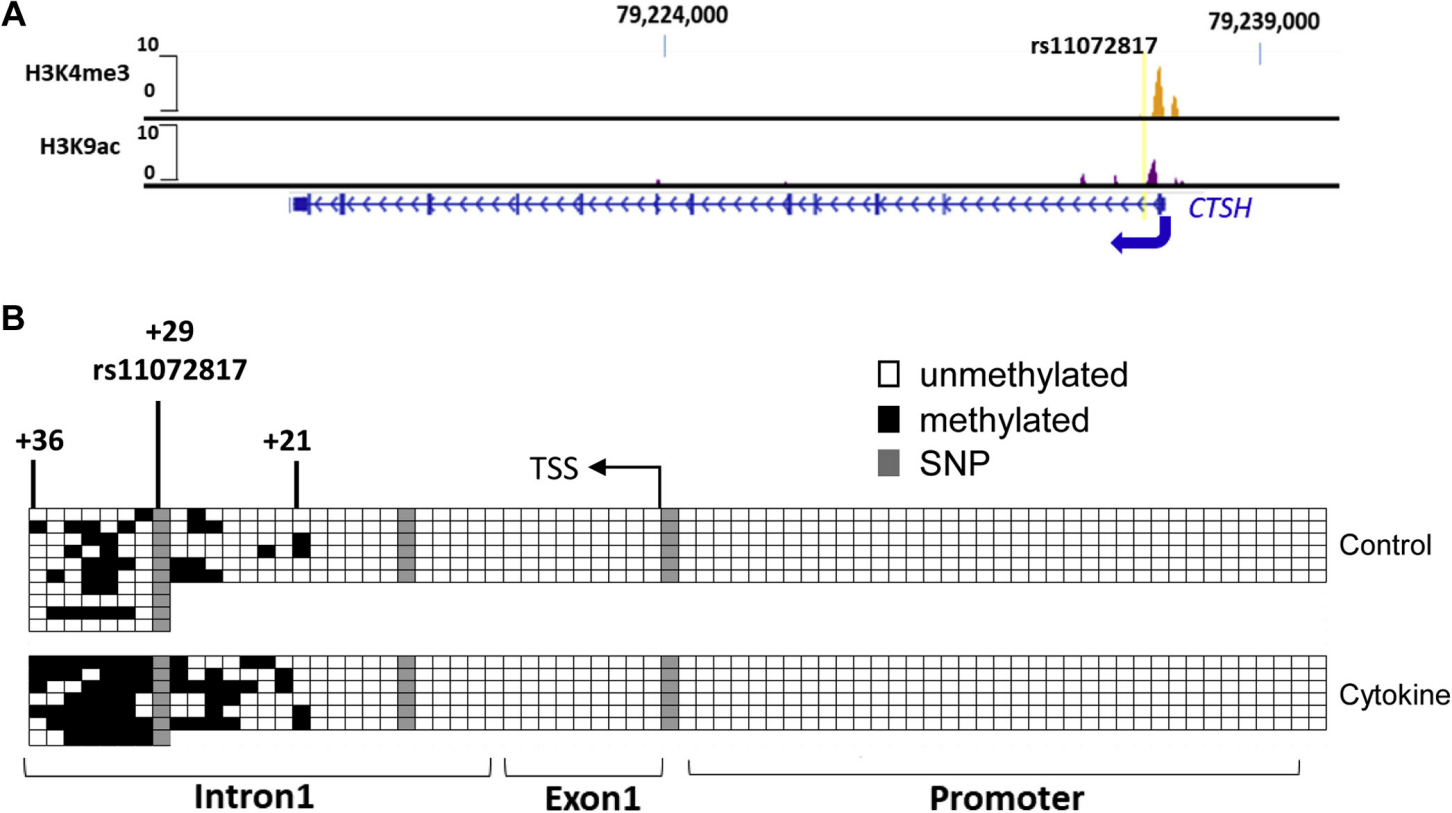
**An open chromatin region in intron 1 of *CTSH* became hypermethylated in human islets after IL-1β + TNF-α + IFN-γ exposure for 24 h.***A*, using the reference data from the National Institutes of Health Roadmap Epigenomics Mapping Consortium, H3K4me3 and H3K9ac peaks identified an open chromatin overlapping the intron 1 of *CTSH*. *B*, bisulfite sequencing of human islets derived from a nondiabetic individual with/without the exposure of the proinflammatory cytokine cocktail; ten clones for the control and seven clones for the cytokine-treated DNA sample were sequenced. *Black squares* represent methylated CpGs, and *white squares* represent unmethylated CpGs. SNP-CpGs were highlighted in *gray*. SNP rs11072817 overlaps with CpG29, for which DNA methylation status was excluded from the analysis. CpG, cytosine–phosphate–guanine dinucleotide; *CTSH*, cathepsin H; IFN-γ, interferon γ; IL-1β, interleukin 1β; TNF-α, tumor necrosis factor α.

Purified pancreatic islets incubated with a cytokine cocktail containing IL-1β + TNF-α + IFN-γ for 24 h revealed a marked reduction in *CTSH* mRNA expression levels (*p* < 0.0001, n = 10) accompanied by upregulation of proinflammatory cytokine signature genes, such as *IRF-1* (*p* = 0.0286, n = 4), *ICE* (*p* = 0.0286, n = 4), *FAS* (*p* = 0.0286, n = 4), and *BBC3* (*p* = 0.0286, n = 4) (Fig. 1). To test whether these cytokines regulate *CTSH* transcription by modifying its DNA methylation, we first searched for open chromatin in *CTSH* suggestive for epigenetic regulation. Using reference data from the National Institutes of Health Roadmap Epigenomics Mapping Consortium (15), we found that in normal human islets, the *CTSH* promoter and intron 1 regions overlapped with H3K4me3 and H3K9ac peaks (Fig. 2*A*), indicating open chromatin for active transcriptional regulation. The *CTSH* promoter and intron 1 regions are CG rich, and therefore, bisulfite sequencing was performed to examine whether cytokine exposure of islets modified *CTSH* DNA methylation in this region. We found that upon cytokine exposure, the methylation status of the *CTSH* promoter (−272 bp to −1 bp upstream of the transcription start site [TSS]) was unchanged, whereas a region in intron 1 containing CpGs 21–36 (+371 bp to +807 bp downstream of TSS, the first CpG site downstream of TSS is denoted as CpG1) was hypermethylated with the average methylation increased by 11% to 46% (n = 3, Figs. 2*B* and S1).

### Methylation of three intron 1 CpG sites attenuates promoter activity

**Figure 3:**
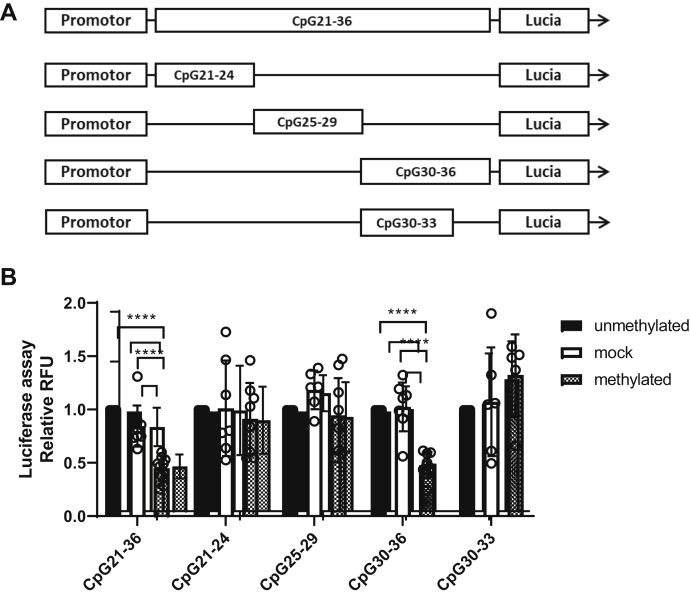
***In vitro* methylation of *CTSH* intron 1 CpG34–36 downregulates the promoter activity of a CpG-free luciferase plasmid.***A,* CpG21–36, CpG21–24, CpG25–29, CpG30–36, or CpG30–33 were inserted downstream of a generic promoter into a CpG-free luciferase plasmid. *B,* plasmids were *in vitro* methylated by M. SssI or mock methylated before transfected into HEK293 cells. Luciferase activities were compared between the unmethylated, mock-methylated, and methylated plasmids. Three independent experiments were conducted. CpG, cytosine–phosphate–guanine dinucleotide; *CTSH*, cathepsin H; HEK293, human embryonic kidney 293 cells.

To investigate whether DNA methylation of the open chromatin region in intron 1 mediates transcriptional downregulation, CpG21–36 were cloned into a CpG-free luciferase vector (pCpG-lucia), downstream of an elongation factor 1α (EF-1α) promoter (Fig. 3*A*). Subsequently, the plasmids were either *in vitro* methylated by M. SssI or mock methylated. Methylation status of CpG21–36 was confirmed by digesting plasmids using methylation-sensitive restriction enzymes (data not shown). Transient transfection in human embryonic kidney 293 (HEK293) cells showed that *in vitro* methylation of CpG21–36 significantly downregulated the EF-1α promoter activity compared with the mock-methylated plasmid or the unmethylated control (*p* < 0.0001, n = 3; Fig. 3*B*). To pinpoint the exact CpG sites within intron 1 that are responsible for regulating *CTSH* transcription, unmethylated, methylated, or mock-methylated plasmids containing CpG21–24, CpG25–29, CpG30–36, and CpG30–33 were analyzed separately for the effect of their methylation on promoter activity. Our results showed that only the methylation of CpG30–36 attenuated the generic promoter activity by reducing the luciferase signal by approximately 50% (*p* < 0.0001, n = 3; Fig. 3*B*). Promoter activity was however not affected by CpG30–33 methylation. Taken together, these data suggested that methylation of the CpG34–36 site in intron 1 is responsible for promoter downregulation.

### *CTSH* intron 1 hypermethylation is caused by downregulation of *Tet1*/*Tet3*

**Figure 4:**
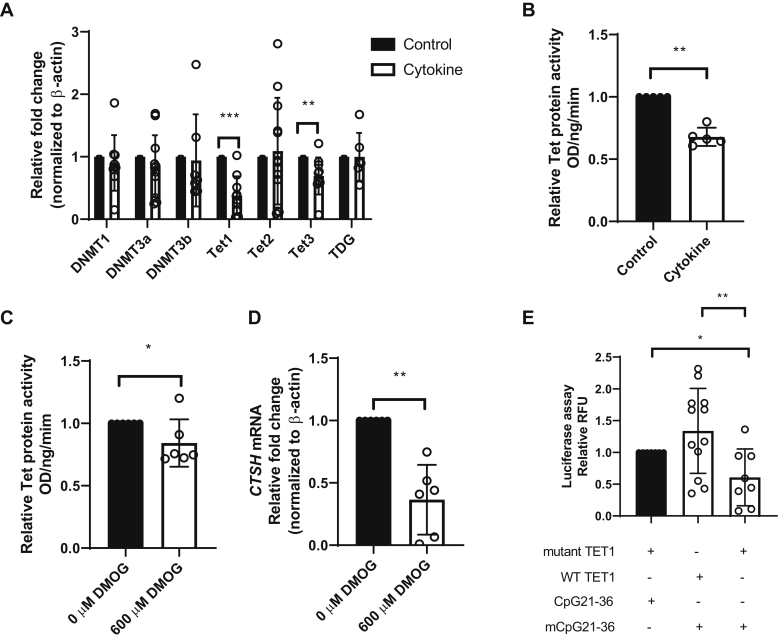
**Inhibition of Tet enzymes reduces *CTSH* transcription.***A*, relative gene expression levels of DNA methyltransferases *DNMT1* (n = 9), *DNMT3a* (n = 11), *DNMT3b* (n = 7), DNA demethylases *Tet1* (n = 10), *Tet2* (n = 12), *Tet3* (n = 10), as well as *TDG* (n = 6) in control islets and islets treated with IL-1β + TNF-α + IFN-γ; expression levels were normalized to β-actin and arbitrarily set to one in control islets. *B*, relative Tet protein activities in cytokine-treated and control human islets (n = 5). *C*, relative Tet protein activities in 48-h DMOG-treated and control human islets (n = 6). *D*, relative *CTSH* mRNA levels (normalized to β-actin) in 48-h DMOG-treated and control human islets (n = 6). *E*, relative luciferase activity of CpG21–36 luciferase plasmid when overexpressing Tet1 in HEK293 cells. Unmethylated (CpG21–36) or methylated (mCpG21–36) luciferase plasmid carrying the *CTSH* intron 1 CpG sites were cotransfected either with the plasmid expressing the functionally active Tet1 catalytic domain or the plasmid expressing the mutant inactive Tet1 catalytic domain at a ratio of 1:6. *CTSH*, cathepsin H; DMOG, dimethyloxalylglycine; HEK293, human embryonic kidney 293 cells; IFN-γ, interferon γ; IL-1β, interleukin 1β; Tet, 10–11 translocation; TNF-α, tumor necrosis factor α.

DNA methylation is a dynamic process. Methyl group is added to the 5-cytosine by DNA methyltransferases (DNMTs), and 5-mC is oxidized by 10–11 translocation (Tet) proteins to generate 5-hydroxymethylcytosine, 5-formylcytosine, and 5-carboxylcytosine (16). 5-formylcytosine and 5-carboxylcytosine are subsequently excised by thymine DNA glycosylase (TDG) back to 5-cytosine (16). Therefore, hypermethylation can result from either upregulation of DNMTs or downregulation of Tet or TDG enzymes. To investigate this, first, we verified the expression of *Tet* in purified human islets. Using quantitative RT–PCR, we found that the Ct values of *Tet*1, 2, 3 were approximately 25, 23, and 26, respectively, against the Ct value of approximately 15 for *β-ACT* (data not shown). The expression of *Tet* was also supported by other studies using primary human islets as well stem cell–differentiated beta cells (17, 18, 19). Following a 24-h proinflammatory cytokine exposure, the expression of DNMTs *DNMT1* (*p* = 0.22, n = 9), *DNMT3a* (*p* = 0.057, n = 11), *DNMT3b* (*p* = 0.18, n = 7), *Tet2* (*p* = 0.4796, n = 12), and *TDG* (*p* = 0.36, n = 6) did not change, but the expression of *Tet1* and *Tet3* was significantly reduced (*p* = 0.0006, n = 10 and *p* = 0.0006, n = 10, respectively, Fig. 4*A*). Functionally, cytokine exposure significantly reduced Tet hydroxylase activities (*p* = 0.0079, n = 5, Fig. 4*B*). Reduction of Tet1 in nuclear lysate after cytokine exposure was seen using Western blotting (Fig. S2). These suggest that *CTSH* hypermethylation was caused by attenuated CpG demethylation by Tet proteins. To further test this hypothesis, we examined whether Tet protein inhibition by a broad dioxygenase inhibitor dimethyloxalylglycine (DMOG) lowered *CTSH* transcription (20). After testing a range of DMOG concentrations (100, 300, and 600 μM) and incubation times (24 and 48 h), we decided to incubate human islets with a DMOG concentration of 600 μM for 48 h, where no apparent changes in cell morphology were found under microscopic examination. Treatment with DMOG caused a significant reduction in Tet enzyme activity (*p* = 0.0476, n = 6; Fig. 4*C*). Treatment with DMOG (600 μM × 48 h) resulted in a significant suppression of *CTSH* transcription (*p* = 0.0022, n = 6; Fig. 4*D*) supporting the hypothesis that cytokines downregulate *CTSH* transcription by suppressing Tet activity resulting in hypermethylation of the CpG region in intron 1 we identified. To test whether overexpressing Tet1 can demethylate *CTSH* intron 1 CpGs, we cotransfected a plasmid expressing the human Tet1 catalytic domain together with the *in vitro* methylated CpG21–36 lucia plasmid into HEK293 cells. Figure 4*E* shows that only the Tet1 functional catalytic domain (*p* = 0.009, Welch's test) but not the mutant Tet1 catalytic domain can reactivate the expression of the methylated CpG21–36 plasmid.

### The T1D protective allele correlates with higher methylation variability than the risk allele

**Figure 5:**
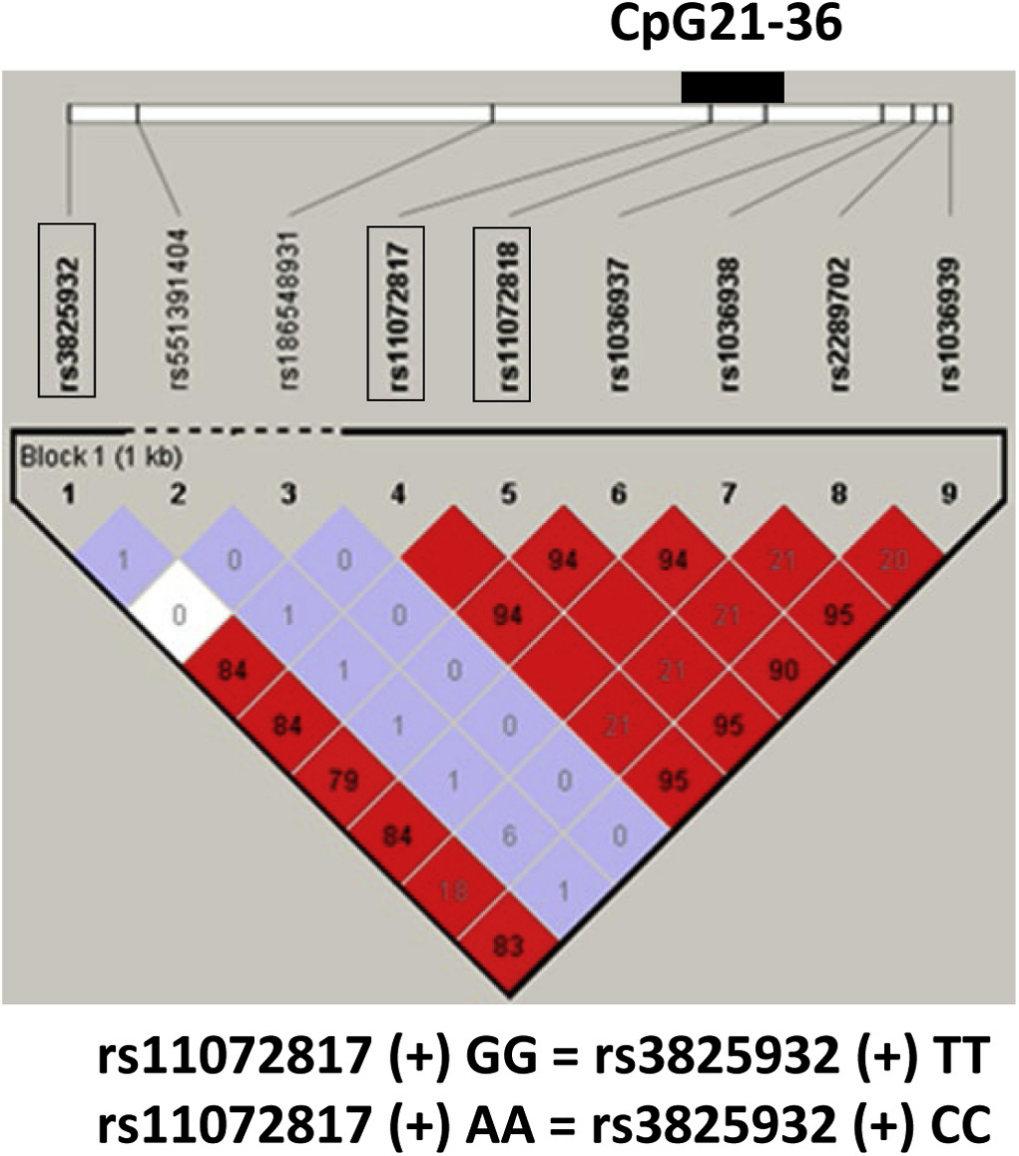
**Linkage disequilibrium relationship between a T1D lead variant SNP rs3825932, SNP rs11072817, and SNP rs11072818 in the Northern European population.** Genetic data were retrieved from the 1000 Genomes phase 3 data. rs11072817 and rs11072818 were located within the hypermethylated region in intron 1 (CpG21–36). Linkage disequilibrium map was plotted using Haploview program. Numbers represent the *r*^2^ values between two SNPs. T1D, type 1 diabetes.

**Figure 6:**
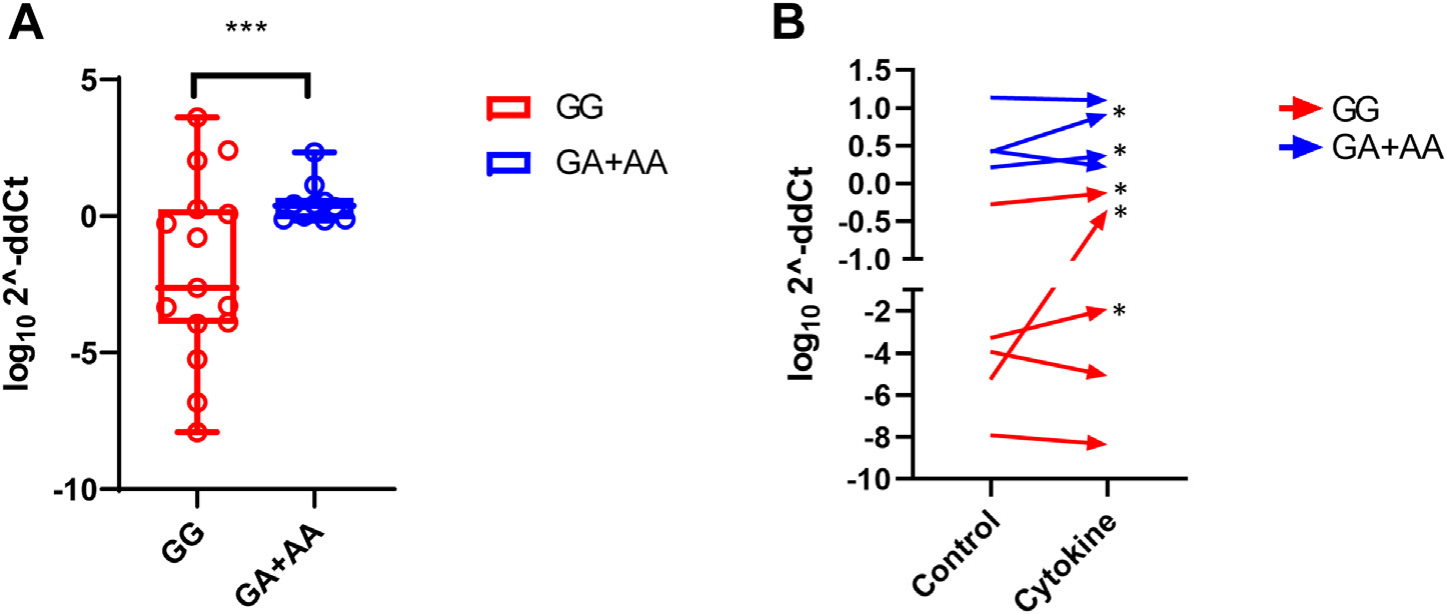
**The G allele of SNP rs11072817 correlated with increased methylation variability at CpG34.***A*, fifteen individuals from the GG group and ten individuals from the GA + AA group were quantified by MS-qPCR. CpG34 methylation levels were divided by rs11072817 genotypes. *Boxplots* represent interquartile ranges; *lines* within the boxplots represent medians, *whiskers* represent minimum–maximum methylation levels. The G allele correlates with increased methylation variability than the A allele (*F* test for variances, *p* < 0.0001, denoted as ∗∗∗ in the graph). *B*, nine pairs of islet samples treated or not treated with cytokines were analyzed by MS-qPCR. *Arrows* represent paired methylation change. Five of nine pairs showed increase in CpG34 methylation after cytokine addition, as marked by *asterisks*. Genotypes were highlighted in *red* and *blue*. CpG, cytosine–phosphate–guanine dinucleotide; MS-qPCR, methylation-specific quantitative PCR.

To investigate whether the risk variant of *CTSH* affects the DNA methylation of CpG34–36, we first conducted linkage disequilibrium (LD) analysis using the 1000 Genomes phase 3 data. We found that SNP rs3825932, a GWAS tagged T1D SNP in the Northern European population (*r*^2^ = 0.84; Fig. 5), is in tight LD with SNPs rs11072817 and rs11072818 that are located within the cytokine-induced hypermethylated region in intron 1. From hereon, we analyzed only rs11072817 because it is in complete LD with rs11072818. To investigate the relationship between genotype and *CTSH* intron 1 methylation in humans, we developed a Taqman methylation-specific quantitative PCR (MS-qPCR) assay that selectively quantifies CpG34 methylation in the presence of unmethylated DNA background (Fig. S3). Using MS-qPCR, we found that the protective variant G allele of rs11072817 (GG; n = 15) correlated with a significantly higher methylation variability compared with the A allele (GA + AA; n = 10) in normal islets (*F* test for variances, *p* < 0.0001; Fig. 6*A*). There tends to be a lower mean methylation in individuals with the G allele than those with the A allele (Welch's *t* test, *p* = 0.016). We also examined CpG34 methylation profile in nine pairs of islet samples treated or not treated with cytokines. Five of nine pairs showed increase in CpG34 methylation (Fig. 6*B*), which were found in both the GG and GA + AA genotypes.

### The T1D protective allele correlates with lower mean *CTSH* expression with and without cytokine treatment

**Figure 7:**
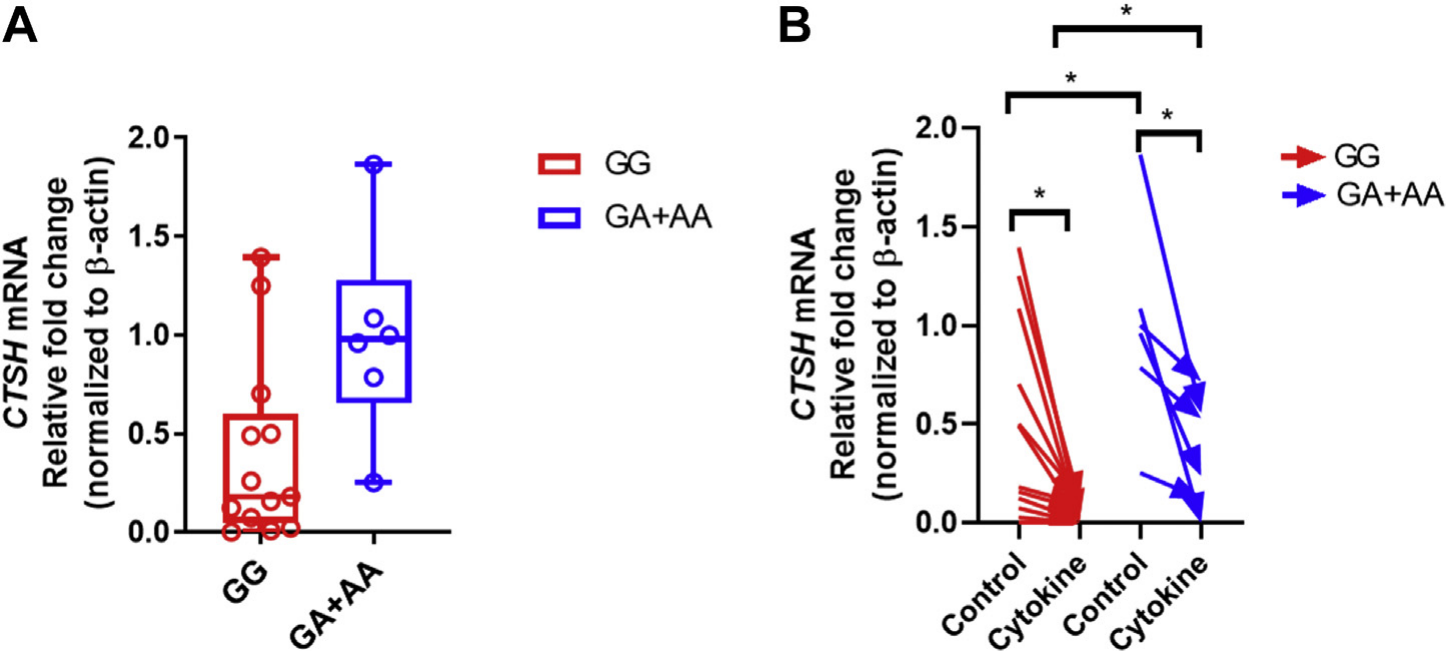
**Relationship between rs11072817 genotypes and *CTSH* expression levels in nondiabetic normal and proinflammatory cytokines IL-1β + TNF-α + IFN-γ–treated human islets.***A*, in normal islets, the G allele of rs11072817 (n = 13) correlated with a lower mean *CTSH* expression than the A allele (n = 6) (*p* = 0.04; statistics were not shown in the graph). *Boxplots* present interquartile ranges; *lines* within the boxplots represent median gene expression levels; *whiskers* represent minimum–maximum expression levels. *B*, *CTSH* transcription was compared in 18 pairs of islet samples (GG, n = 12; GA + AA, n = 6) treated or not treated with cytokines. Addition of cytokines reduced *CTSH* transcription (GG, *p* = 0.02; GA + AA, *p* = 0.03), but individuals with the GG genotype had a lower mean *CTSH* expression than those with the GA + AA genotype even after cytokine addition (*p* = 0.02). *CTSH*, cathepsin H; IFN-γ, interferon γ; IL-1β, interleukin 1β; TNF-α, tumor necrosis factor α.

Transcriptional variability was not detected between the rs11072817 genotypes in samples that we analyzed (*F* test for variances, *p* = 0.68; GG, n = 13; and GA + AA, n = 6; Fig. 7*A*). However, extraction of the Genotype-Tissue Expression (GTEx) consortium data from 305 human pancreases showed that the G allele correlated with higher transcriptional variability (larger interquartile range) than the A allele (Fig. S4). Of the 19 samples collected, we compared transcription in 18 pairs of islets treated or not treated with cytokines (GG, n = 12; GA + AA, 6). In normal islets, the mean expression in islets carrying the G allele was lower than that of the A allele (*p* = 0.04, Welch's test; Fig. 7, *A* and *B*), which was consistent with the pancreas expression quantitative trait locus data of the GTEx consortium (Fig. S4). Under proinflammatory cytokines IL-1β + TNF-α + IFN-γ treatment, C*TSH* expression was reduced in both genotypes, but individuals with the G allele still correlated with a significantly lower expression than those with the A allele (*p* = 0.02, Welch's test; Fig. 7*B*).

## Discussion

In this study, we investigated whether DNA methylation mediates the genetic and environmental influence (act as proinflammatory cytokines) of T1D risk at the *CTSH* locus. We identified a novel mechanism where the genetic risk and proinflammatory cytokines regulate *CTSH* expression by differential DNA methylation of the same CpG residues in intron 1.

*CTSH* encodes cathepsin H, which is a member of the papain-like cysteine proteases that are primarily involved in endolysosomal protein degradation, activation of other proteases (21), as well as major histocompatibility complex class II antigen presentation. Although *CTSH*-null mice do not demonstrate gross developmental defects (22), *CTSH* was associated with autoimmunity in mouse models and humans including experimental autoimmune encephalomyelitis (23) and T1D (24). *CTSH* is expressed in pancreatic beta cells and antigen-presenting cells but not in T cells (25). There is a lack of consensus on how it contributes to the pathogenesis of T1D. Studies found that downregulation of *CTSH* in the presence of proinflammatory cytokines increases beta-cell apoptosis *via* the small GTPase *Rac2* pathways (14, 26), but this effect correlated with the T1D protective allele not the risk allele (14). Here, we showed that *CTSH* downregulation is induced by DNA hypermethylation of three key CpG residues of an open chromatin upon inflammatory cytokine treatment, which was in line with the general consensus that DNA hypermethylation downregulates gene expression.

**Figure 8:**
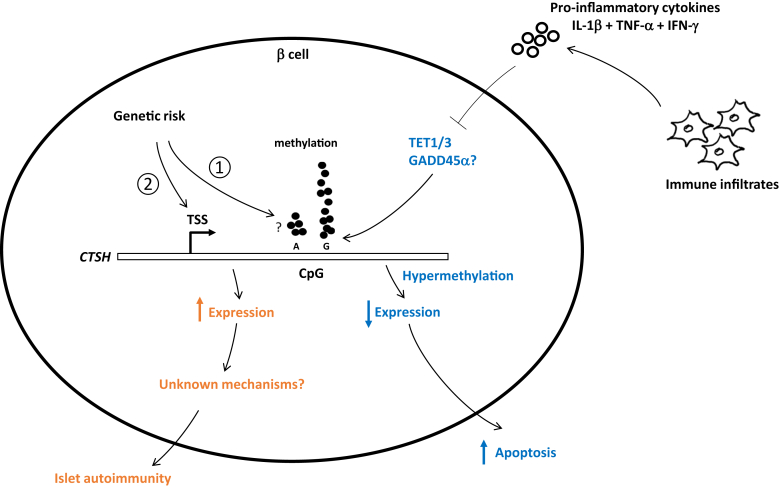
**Potential mechanism for the paradoxical differential effects of genetic and environmental factors on *CTSH* gene and their contribution to the development of T1D.** Individuals with the protective variant (G of rs11072817) have higher DNA methylation and transcriptional variability. They are more sensitive to immune-mediated proinflammatory cytokines. In the presence of cytokines, *CTSH* intron 1 becomes hypermethylated, and its expression is downregulated. *CTSH* downregulation in beta cells promotes apoptotic cell death. In contrast, the genetic variant (A of rs11072817) correlates with high *CTSH* transcription possibly *via* (1) lowered DNA methylation and (2) other types of regulations. Individuals with the genetic risk variant likely develop T1D early driven by a strong genetic effect *via* unknown mechanisms. *CTSH*, cathepsin H; T1D, type 1 diabetes.

The uniqueness of our findings is that the T1D risk allele at the *CTSH* locus correlated with less methylation variability at these CpG residues, whereas the protective allele correlated with higher methylation variability. Increased methylation variability was found in affected siblings of T1D and rheumatoid arthritis discordant-monozygotic twin pairs (27, 28), implying that epigenetic plasticity potentially mediates the disease-causing environmental stimuli. Similarly, substantial methylation and transcriptional variability were identified in neutrophils rather than monocytes and T cells because neutrophils are the first responders to inflammatory stimuli (29). Higher phenotypic plasticity renders cells a more rapid and effective response to environmental cues. For example, B cells and T cells use recombination to generate a highly diverse repertoire of immunoglobulins and T-cell receptors. Thus, our findings support the notion that individuals with the protective variant of *CTSH* were more sensitive to proinflammatory cytokines–induced beta-cell damages (14). In contrast, individuals with the risk variant may develop T1D driven by a strong genetic effect *via* a different mechanism (Fig. 8) (30). Recently, a variant of the coxackie and adenovirus receptor (*CXADR*) gene rs6517774 was associated with islet autoimmunity in young children (31), but this variant was not identified in the T1D GWAS study. Together with our findings, these results imply that individuals with non-GWAS risk alleles may respond to environmental factors, leading to beta-cell damages.

We found that *CTSH* intron 1 hypermethylation is dependent on the loss of Tet1/3 activities after a 24-h cytokine treatment. Although loss of Tet is considered to cause global hypermethylation, Tet1 has been shown to require growth arrest and DNA damage–45 alpha (GADD45α) and other cofactors as adaptors to function, where GADD45α has locus-specific effect (32, 33, 34). GADD45α is highly expressed in beta cells (18), thus, further studies are necessary to investigate the involvement of GADD45α and other cofactors in recruiting Tet1 to the *CTSH* locus to maintain its transcriptional activation. Our results are different than those reported by Rui *et al*. (35, 36), who found that cytokines IL-1β + TNF-α + IFN-γ increased the transcription of *DNMT3A* and *Tet2* in human islets, whereas we did not. The differences could be due to the different cytokine concentrations used in *ex vivo* culture. A limitation of using *ex vivo* models is that they cannot recapitulate the kinetics of cytokine exposure of islets that happens *in vivo*. Thus, the changes in gene transcription seen in our experiment may represent a cross-sectional and temporal effect, which could be dependent on the cytokine concentration and/or duration of the treatment (37).

Our study has limitations. First, our sample size to estimate the mean and variance of methylation and gene expression was limited because of the availability of human islets. In control samples, we observed that individuals with the GG genotype had a higher methylation variability and a lower mean methylation level. Because of variability, we did not have enough power to calculate an accurate mean in those individuals. We did not observe a significant transcriptional variability between the GG and GA + AA genotypes in our samples, but differences were seen in 305 pancreases from the GTEx consortium data, a sample size large enough to detect differences in transcriptional variability. Second, purity of human islets, age, gender, ethnicity, body mass index, pre-existing medical conditions, and hospital treatments of the donors may have also contributed to some variations of the data (38, 39). Third, we used transient transfection to model the inhibitory effect of *CTSH* intron 1 methylation. This is a limitation because of the potential interassay variability.

In conclusion, our data revealed a novel molecular mechanism whereby differential DNA methylation mediates the genetic and environmental influences at the *CTSH* locus. Given that *CTSH* genetic risk was associated with early diabetes onset (12) and impaired beta-cell function independent of the human leukocyte antigen effect (13), our results highlight the importance of *CTSH* gene dysregulation in the natural history of T1D progression. Such understanding may form the basis for translational studies targeting *CTSH* in T1D.

## Experimental procedures

### Cell culture

Human islets were obtained from the National Institutes of Health–supported Integrated Islet Distribution Program. Islets were received as deidentified anonymous samples. The project was approved by the Albert Einstein College of Medicine Institutional Review Board as exempt and abided by the Declaration of Helsinki principles. Islets (purity, 80–95%; viability, 90–98%) were cultured either in basal medium containing RPMI1640 with 10% fetal bovine serum, 5.5 mM glucose, 1% penicillin–streptomycin (P/S), or in basal medium with proinflammatory cytokines, including 5 ng/ml IL-1β (ab9617; Abcam), 40 ng/ml TNF-α (ab9632; Abcam), and 1000 ng/ml IFN-γ (RINFG100; Thermo Fisher Scientific). Islets were cultured in the presence and absence of cytokines for 24 h before DNA/RNA extraction. For Tet inhibition, DMOG (Sigma–Aldrich; catalog no.: D3695) was dissolved in 1× PBS and added to the culture medium at 600 μM. Islets were cultured with DMOG for 48 h, and medium was refreshed every 24 h. HEK293 cells were routinely cultured in RPMI1640 medium with 10% and 1% P/S. We chose HEK293 to assess the effect of *CTSH* intron 1 methylation because HEK293 is a human cell line, easy to transfect, and expresses human transcription factors sufficient enough to study *CTSH* gene regulation. HEK293 is however not suitable to study the downstream events associated with *CTSH* transcriptional regulation, which might be specific to beta cells. For transient transfection experiments, HEK293 cells were cultured in normal growth medium with no P/S for 24 h before transfection.

### DNA and RNA extraction

Cultured islets (250–500 islet equivalents) were collected by gentle pipetting, and DNA was isolated using the QiaAmp DNA blood mini kit (Qiagen; catalog no.: 51104) according to the manufacturer's recommended protocol. RNA was isolated using the TRIzol reagent (Thermo Fisher Scientific; catalog no.: 15596026). Five hundred nanograms of total RNA were reverse transcribed into complementary DNA using the SuperScript III First-Strand Synthesis System (Thermo Fisher Scientific; catalog no.: 18080051) according to the manufacturer's protocol.

### Real-time qPCR, Taqman genotyping, and Sanger sequencing

Complementary DNA was amplified using the SYBR Green PCR Master Mix (Applied Biosystems; catalog no.: 4309155) together with gene-specific primers (Table S1). Real-time qPCR was performed on a QuantStudio 3 Real-time qPCR system (Applied Biosystems) using the relative quantification 2^−(ΔΔCt)^ method. All PCRs were conducted at an annealing temperature of 58 °C for a total of 55 cycles. PCR amplicon specificity was assessed using the melting curve analysis. Relative abundance of gene expression was normalized against the beta-actin (*ACTB*) housekeeping gene. SNP genotyping for rs3825932 was performed using the Taqman allele discrimination assay (Applied Biosystems; catalog no.: 4351379). Sanger sequencing (GENEWIZ) was used to determine the genotype for SNP rs11072817 (for primer sequences, see Table S1)

### Bisulfite Sanger sequencing

For bisulfite Sanger sequencing, 500 nanograms of DNA per sample were bisulfite treated using the EZ DNA Methylation-Direct Kit (Zymo; catalog no.: D5020) following the manufacturer's protocol. Bisulfite-converted DNA was then amplified using methylation-insensitive sequencing primers (Table S1) on a T100 Thermal Cycler (Bio-Rad) for 55 cycles. PCR products were ligated into pCR 2.1 vectors using the Original TA Cloning Kit (Invitrogen; catalog no.: K202020) and transformed into DH5α Competent *Escherichia coli* cells (Thermo Fisher Synthetic; catalog no.: 18265017). Selected colonies were grown overnight, DNA was extracted using the QIAprep Spin Miniprep kit (Qiagen; catalog no.: 27104) and sequenced using Sanger sequencing. Five to eight clones per DNA sample were sequenced. Percentage of methylation was determined by averaging DNA methylation levels of all the sequenced clones.

### Site-directed mutagenesis

A DNA sequence spanning an intron 1 region of *CTSH* (chr15: 79,236,578–79,237,097, hg19) was cloned into the intron of the pCpG-free-lucia plasmid (Invivogen; catalog no.: pcpgf-promlc), which was denoted as pCpG-free-CTSH intron 1-lucia plasmid thereafter. The expression of the lucia gene was driven by a modified human EF-1α promoter. The modified EF-1α promoter in the pCpG-free-lucia plasmid was devoid of CpG sites and therefore was ideal for this experiment because it allowed us to only methylate the *CTSH* intron 1 without getting methylated itself. In contrast, the *CTSH* promoter is enriched with CpG sites. *CTSH* promoter would be methylated when the plasmid is *in vitro* methylated and therefore could not be used for this experiment. To truncate CpGs within this region, site-directed mutagenesis primers were designed using the NEBaseChanger tool (New England Biolabs). Site-directed mutagenesis was performed using the Q5 site-directed mutagenesis kit (New England Biolabs; catalog no.: E0554S) following the manufacturer's protocol. pCpG-free-CTSH intron 1-lucia plasmids were transformed into the ChemiComp GT115 *E. coli* cells (Invivogen; catalog no.: gt115-11). Selected colonies were grown overnight for DNA extraction.

### *In vitro* methylation and luciferase assay

To methylate the pCpG-free-CTSH intron 1-lucia plasmids, greater than 2 μg plasmid DNA per sample was treated using 40 U CpG methyltransferase M. SssI (New England Biolabs; catalog no.: M0226M) in a 50 μl reaction mixture for 2 h. After the first round of treatment, plasmid DNA was methylated for a second round in a 100 μl reaction mixture for 2 h. For mock-methylated plasmid (negative control), distilled H_2_O was added into the reaction mixture instead of M. SssI. To verify the success of *in vitro* methylation, unmethylated and methylated plasmids (300 ng) were then digested using the methylation-sensitive restriction enzyme HpaII or the methylation-insensitive restriction enzyme SatI. For transient transfection, unmethylated, mock methylated, and methylated pCpG-free-CTSH intron 1-lucia plasmids (50 ng per well) were transfected into HEK293 cells using the Lipofectamine 3000 reagent (Thermo Fisher Scientific; catalog no.: L3000001) for 24 h at a cell density of 3.5 × 10^4^ cells/well. pGL4.10-firefly plasmids containing the *CTSH* promoter (100 ng per well) were simultaneously transfected as transfection controls. For Tet1 cotransfection experiment, a plasmid expressing either the functionally active Tet1 catalytic domain (180 ng; Addgene; catalog no.: 124082) or the mutant inactive Tet1 catalytic domain (180 ng; Addgene; catalog no.: 124083) was cotransfected with either the unmethylated or the methylated pCpG-free-CTSH intron 1-lucia plasmid (30 ng), as well as the pGL4.10-firefly transfection control plasmid (100 ng). Luciferase activity was measured using the Dual-luciferase reporter assay (Promega; catalog no.: E1910). Relative luciferase activity for each construct was calculated by normalizing lucia activity to firefly activity. Luciferase signal for the mock methylated and methylated constructs were then normalized to the unmethylated counterparts, so that the expression of the unmethylated plasmids was arbitrarily set to one.

### Tet hydroxylase activity quantification

Protein lysates were extracted from islets (∼1500 islet equivalents per treatment) using radioimmunoprecipitation assay buffer with proteinase inhibitor, then sonicated for three times at 4 kHz for 5 s each, and pulse vortexed for 30 min prior to centrifugation. Protein concentration was quantified using the bicinchoninic acid assay (Pierce, Thermo Fisher; catalog no.: 23225). Tet hydroxylase activity (10 μg) per sample was measured using the Tet Hydroxylase Activity Quantification Kit (Abcam; catalog no.: ab156913) following the manufacturer's protocol.

### Western blotting

Cytoplasmic and nuclear protein lysates were extracted from human islets (∼1500 islet equivalents per treatment) using the NE-PER Nuclear and Cytoplasmic Extraction kit (Thermo Fisher Scientific; catalog no.: 78833). Cytoplasmic (46 μg) and nuclear lysate (16 μg) were separated using the Mini-PROTEAN TGX gel (Bio-Rad; catalog no.: 4561084) and transferred to nitrocellulose membrane. Membrane was blocked overnight using 1:2 diluted Odyssey tris-buffered saline blocking buffer (LI-COR; catalog no.: 927-50000). Tet1 was detected using the primary rabbit anti-hTET1 antibody (GeneTex; catalog no.: GTX124207) and secondary anti-rabbit IRDye 680RD antibody (LI-COR; catalog no.: 926-68071). Beta-actin was detected using the primary mouse anti-hACTB antibody (Cell Signaling; catalog no.: 8H10D10) and the secondary antimouse IRDye 800CW antibody (LI-COR; catalog no.: 926-32210). Membrane was imaged using the Odyssey Fc imaging system (LI-COR).

### MS-qPCR

For the *CTSH* methylation quantitation assay, forward and reverse methylation-unspecific primers were designed using the Zymo Bisulfite online design tool (https://www.zymoresearch.com/pages/bisulfite-primer-seeker). Methylation-specific Taqman Minor Groove Binding probe was designed using the Primer Express 3.0 software (Applied Biosystems). Briefly, CpG35 was placed at the 5’ end of the probe, and CpG34 was placed at the 3’ end of the probe. Because 3’ end of the probe determines the most specificity, the assay primarily quantifies methylation of CpG34. Forward reverse primers and the Taqman probe (Fluorescein-Minor Groove Binding) each at the final concentration of 300 nM were used.

For the *ACTB* assay, primers and probe (Fluorescein-Carboxytetramethylrhodamine) were designed using the Primer Express 3.0 software (Applied Biosystems). Primers and probe do not discriminate methylation status of the *ACTB* DNA; therefore, *ACTB* serves as a housekeeping gene control. Three hundred nanomolars for each primer and probe was used. Primer and probe sequences for both the *CTSH* and *ACTB* MS-qPCR assay were listed in Table S1.

A touchdown PCR approach was used to perform the amplification. MS-qPCR was initially conducted at the annealing temperature 57 °C for five cycles to promote primer/probe binding but not to record the fluorescent data and then at 55 °C for 50 cycles to record the fluorescent data. *CTSH* methylation was quantified using the ΔΔC_t_ method. ΔΔC_t_ for each sample was calculated using the ΔCt of the sample minus the ΔC_t_ of a fully methylated DNA standard (Qiagen, EpiTech; catalog no.: 59695), expressed as ΔΔC_t_ = (C_t_ of *CTSH* − C_t_ of *ACTB*)_Sample_ − (C_t_ of *CTSH* − C_t_ of *ACTB*)_Standard_. Finally, fold change (2^−ΔΔCt^) was log10 transformed to follow a normal distribution.

### Statistical analyses

All data were presented as mean ± SD. Mann–Whitney *U* test was used to compare gene expression levels and protein activities between treated and untreated samples. *F* test was used to compare the variances between individuals with different genotypes. Welch's *t* test was used to compare the sample means with unequal variances. Prism version 7.03 software (GraphPad) was used to conduct statistical analyses. A *p* value ≤0.05 was considered significant.

## Data availability

All data and resources generated during the current study are included in the published article and supporting information.

## Supporting information

This article contains supporting information.

## Conflict of interest

The authors declare that they have no conflicts of interest with the contents of this article.
