# Supplementary material for: Genetic and environmental factors regulate the type 1 diabetes gene CTSH via differential DNA methylation
Source: J Biol Chem. 2021 May 14;296:100774. doi: 10.1016/j.jbc.2021.100774 (PMC8191311; doi:10.1016/j.jbc.2021.100774)
Supplement: Figures S1 to S4 and Table S1 [file mmc1.pdf]

Figure S1

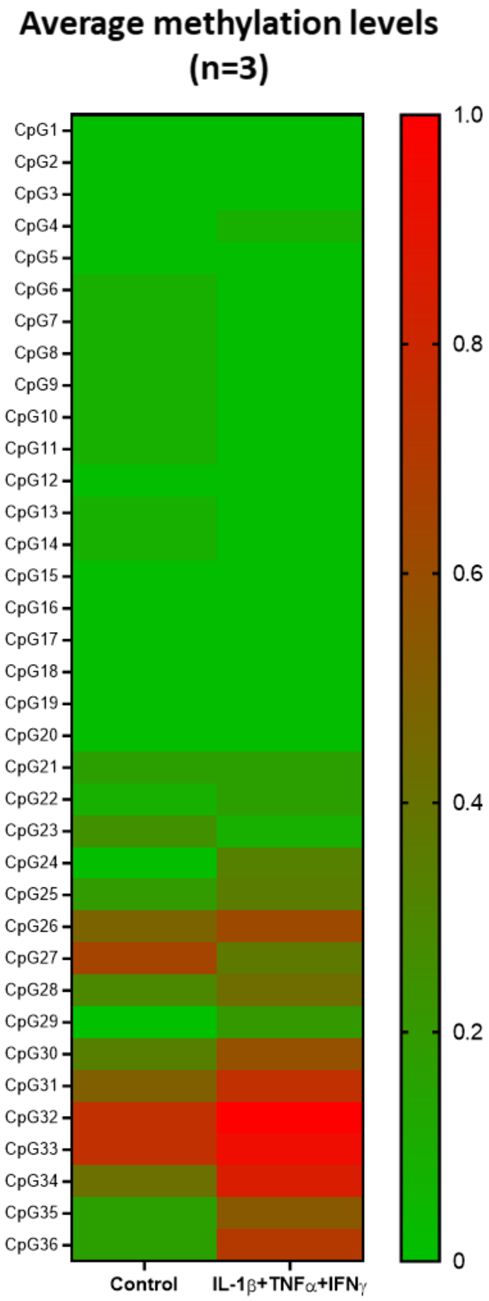

Figure S1: Islet DNA methylation levels of *CTSH* intron1 CpG sites downstream of the transcriptional start site (TSS) exposed with and without pro-inflammatory cytokines IL-1β + TNF-α + IFN-γ for 24 hours. The first CpG downstream of the TSS is denoted as CpG1. Average methylation levels were taken from at least 6 clones per CpG site. Data were collected from sequencing 3 individuals.

Figure S2

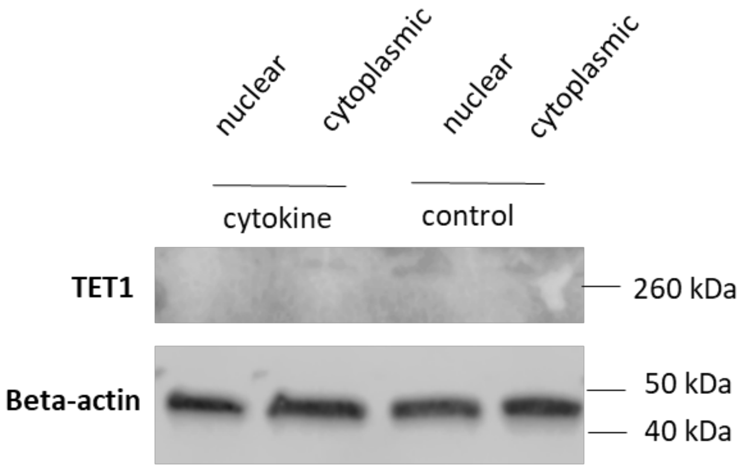

Figure S2: Western blot showing the reduction of Tet1 protein in human islet nuclear lysate after pro-inflammatory cytokine IL-1β + TNF-α + IFN-γ treatment. One thousand five hundred human islet equivalents were treated or not treated with pro-inflammatory cytokine IL-1β + TNF-α + IFN-γ treatment for 24 hours. Cytoplasmic (46μg) and nuclear (16 μg) fraction of the protein lysate from primary human islets were examined in the control and cytokine treated conditions. Human Tet1 (235KDa) and beta-actin (45KDa) bands were indicated.

Figure S3

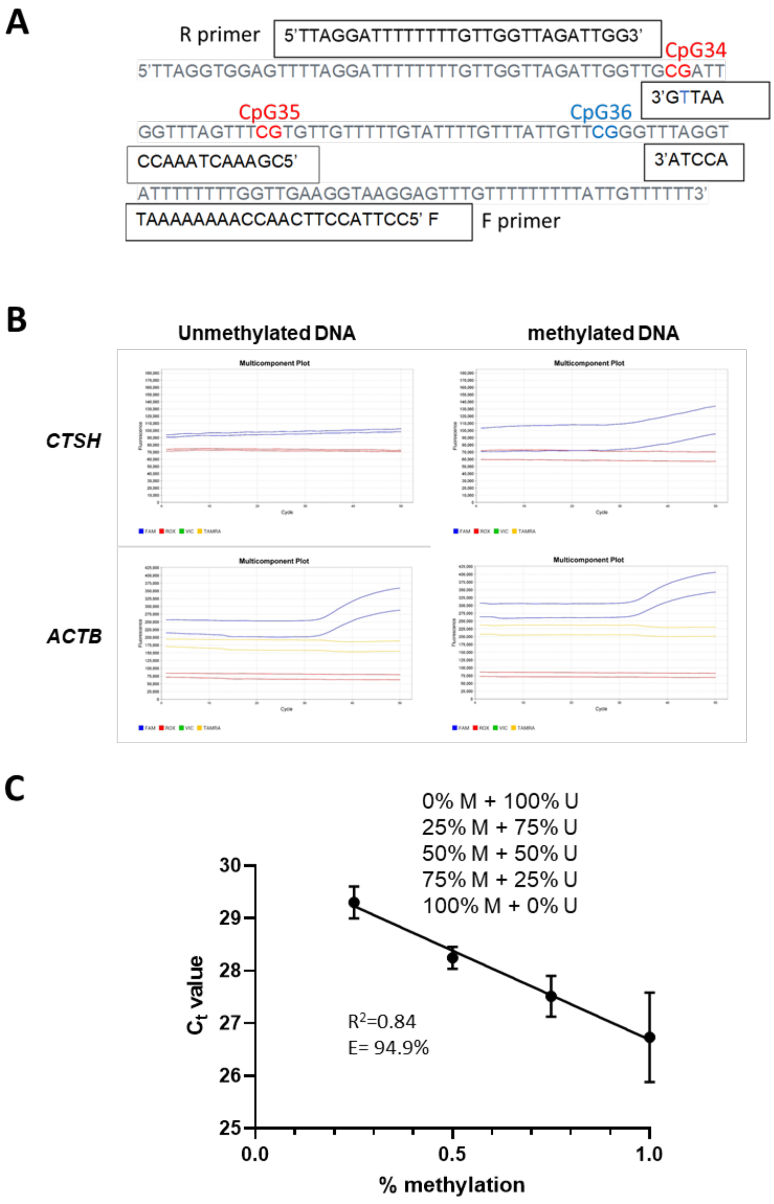

Figure S3: Assay design of the *CTSH* intron1 CpG34 methylation-specific qPCR (MS-qPCR). A, methylation unspecific primers and methylation specific Taqman probe were designed to amplify the region containing *CTSH* intron1 CpG34, C35, and CpG36. The probe was designed to bind CpG34 and CpG35, but CpG36 was unable to be interrogated; an artificial mismatched nucleotide of the probe is highlighted in blue; B, the *CTSH* MS-qPCR assay selectively amplifies fully methylated but not fully unmethylated *CTSH* gene, whereas the *ACTB* qPCR assay does not discriminate the methylation status of the beta-actin gene, C, standard curve showing the PCR amplification efficiency (E=94.9%) using different percentages of methylated DNA (M) when mixed with unmethylated (U) DNA in a total of 20ng per qPCR reaction.

Figure S4

G allele with increased

CTSH expression variability

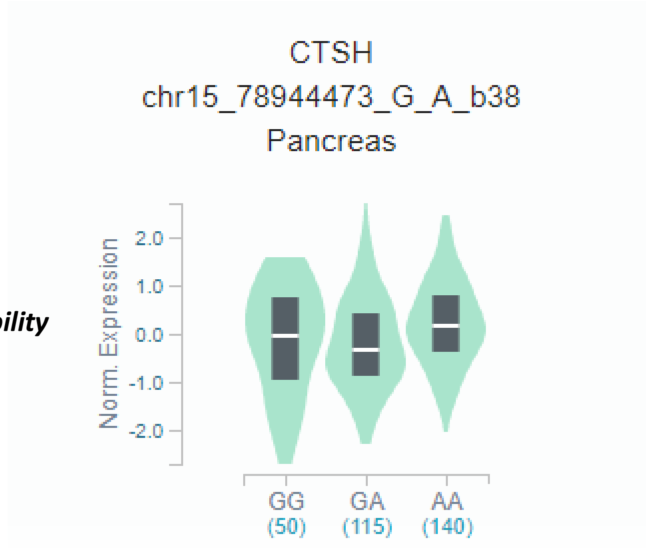

Figure S4: *CTSH* expression levels and their relationship with SNP rs11072817 genotypes in human pancreases. Data were extracted from the GTEx consortium, where 305 human pancreases were analyzed by RNA-seq. Green shaded violin plots represent the density distributions of the normalized *CTSH* expression levels. White lines within the black boxplots represent median expression levels and boxplots represent interquartile ranges. Blue highlighted number under each genotype represents the number of pancreas samples analyzed in each group.

Table S1: qPCR primers, bisulfite sequencing primers, and MS-qPCR primer and probes used in the study.

cDNA qPCR primers		
ACTB	5’GATGAGATTGGCATGGCTTTT3’	5’CACCTTCACCGTTCCAGTTT3’
CTSH	5’TGGCTGTTGGGTATGGAGAAA3’	5’CGATGAGGAAGTACCCGTTCA3’
IRF	5’AGTGTACCGGATGCTTCCACCTC3’	5’TGCTCTTAGCATCTCGGCTG3’
ICE	5’AAATCTCACTGCTTCGGACATGA3’	5’GGAACGTGCTGTCAGAGGTCTT3’
FAS	5’GACTCAGAACTTGGAAGGCCTG3’	5’CATCCCCATTGACTGTGCAG3’
SOCS2	5’GAGCTCGGTCAGACAGGATG3’	5’AGGTAGTCTGAATGCGAGCTATCT3’
BBC3	5’GGACGACCTCAACGCACAGTA3’	5’CATGATGAGATTGTACAGGACCCT3’
DNMT1	5’CAGAAAAGGAATGTGTGAAGGA3’	5’TTGGTTTCCAAGTCACATAACTG3’
DNMT3a	5′CCGATGCTGGGGACAAGAAT3’	5′GTTTCCCCCACACCAGCTC3′
DNMT3b	5’AGGGAAGACTCGATCCTCGT3’	5’GAGAGTCGCGAGCTTGATCT3’
TET1	5’GCTGCTGTCAGGGAAATCAT3’	5’AATTGGACACCCATGAGAGC3’
TET2	5’GTTAGAAAGGAGACCCGA3’	5’CAGGAGCAAAGGCAAGTA3’
TET3	5’ACTCCGGAGAAGATCAAGCA3’	5’GGACAATCCACCCTTCAGAG3’
TDG	5’CCCCGATATTTTGACCTTCA3’	5’CCAGGTCCAGGGTAATGATG3’
Bisulfite sequencing primers		
CpG1-4	5’TTTTTTTTAGAGGAGATAAGGGAG3’	5’AAAACTCCCAAAAACCAAACC3’
CpG5-24	5’GGTTTGGTTTTTGGGAGTTTT3’	5’CACTCAACCCATCTACTCCTCTAAT3’
CpG25-30	5’GGGTTGAGTGTTTAGGGTTTTATTTA3’	5’ACACAAAAATTCACTATTCCAAACA3’
CpG29-36	5’GGGATTTGGTATATAGGGGATTTT3’	5’AAAAAACAAACTCCTTACCTTCAAC3’
MS-qPCR		
CTSH primers	5’TTAGGATTTTTTTGTTGGTTAGATTGG3’	5’ CCTTACCTTCAACCAAAAAAAATACCTA3’
CTSH probe	5’ FAM-CGAAACTAAACCAATTG – MGB 3’	
ACTB primers	5’TGGTGATGGAGGAGGTTTAGTAAGT3’	5’AACCAATAAAACCTACTCCTCCCTTAA3’
ACTB probe	5’FAM-ACCACCACCCAACACACAATAACAAACACA – TAMRA 3’	
rs11072817 sequencing primers		
rs11072817	5’ GCTGTTGGTCTGAGTGCTTTC3’	5’ATCTGTCCCTGCCAGAGGT3’
